# The effect of phytosulfokine alpha on haploid embryogenesis and gene expression of *Brassica napus* microspore cultures

**DOI:** 10.3389/fpls.2024.1336519

**Published:** 2024-02-15

**Authors:** Špela Mestinšek Mubi, Urban Kunej, Valentin Vogrinčič, Jernej Jakše, Jana Murovec

**Affiliations:** Biotechnical Faculty, University of Ljubljana, Ljubljana, Slovenia

**Keywords:** PSK-α, PSK, haploids, doubled haploids, transcriptome, RNA-seq, mitogenic factor, differentially expressed genes

## Abstract

Microspore embryogenesis (ME) is the most powerful tool for creating homozygous lines in plant breeding and molecular biology research. It is still based mainly on the reprogramming of microspores by temperature, osmotic and/or nutrient stress. New compounds are being sought that could increase the efficiency of microspore embryogenesis or even induce the formation of haploid embryos from recalcitrant genotypes. Among these, the mitogenic factor phytosulfokine alpha (PSK-α) is promising due to its broad spectrum of activity *in vivo* and *in vitro*. The aim of our study was to investigate the effect of PSK-α on haploid embryogenesis from microspores of oilseed rape (*Brassica napus* L., DH4079), one of the most important oil crops and a model plant for studying the molecular mechanisms controlling embryo formation. We tested different concentrations (0, 0.01, 0.1 and 1 µM) of the peptide and evaluated its effect on microspore viability and embryo regeneration after four weeks of culture. Our results showed a positive correlation between addition of PSK-α and cultured microspore viability and a positive effect also on the number of developed embryos. The analysis of transcriptomes across three time points (day 0, 2 and 4) with or without PSK-α supplementation (15 RNA libraries in total) unveiled differentially expressed genes pivotal in cell division, microspore embryogenesis, and subsequent regeneration. PCA grouped transcriptomes by RNA sampling time, with the first two principal components explaining 56.8% variability. On day 2 with PSK, 45 genes (15 up- and 30 down-regulated) were differentially expressed when PSK-α was added and their number increased to 304 by day 4 (30 up- and 274 down-regulated). *PSK*, *PSKR*, and *PSI* gene expression analysis revealed dynamic patterns, with *PSK2* displaying the highest increase and overall expression during microspore culture at days 2 and 4. Despite some variations, only *PSK1* showed significant differential expression upon PSK-α addition. Of 16 ME-related molecular markers, 3 and 15 exhibited significant differential expression in PSK-supplemented cultures at days 2 and 4, respectively. Embryo-specific markers predominantly expressed after 4 days of culture, with higher expression in medium without PSK, while on day 0, numerous sporophyte-specific markers were highly expressed.

## 1 Introduction

Microspore embryogenesis (ME) is an *in vitro* process in which haploid microspores are reprogrammed from gametophytic to sporophytic development through the application of stress treatments. In this process, also known as androgenesis, whole plants develop from male gametes due to the totipotency of the plant cells and the process is therefore also used as a model for studies on plant developmental plasticity (Soriano et al., 2014; Seifert et al., 2016). There are two *in vitro* methods of androgenesis: anther culture and isolated microspore culture. Microspore culture is more commonly used since it prevents the influence of somatic tissue on embryogenesis and the regeneration of heterozygotes from somatic cells. The regenerated embryos can be haploid or can become doubled haploid (DH), either by spontaneous polyploidization during *in vitro* culture or by the application of chromosome doubling agents. The final stage of ME is the regeneration of doubled haploid plants homozygous at all loci, which are of great value for plant breeding and basic research. Complete homozygosity of cells simplifies genome assemblies after sequencing, plant breeding, hybrid seed production, quantitative genetic research and mutation discovery. However, although efficient for some plant species, its success is still highly dependent on the genotype of the plant, and it is not yet suitable for all plant genotypes (Maluszynski et al., 2003; Umehara et al., 2005; Szarejko and Forster, 2007; Touraev et al., 2010).

Rapeseed (*Brassica napus* L., 2n = 4x = 38, AACC) is an allotetraploid species (2n = 38, AACC) formed by natural interspecific hybridization between two diploid species *Brassica rapa* (2n = 2x = 20, AA) and *Brassica oleracea* (2n = 2x = 18, CC) (Lu et al., 2019; Song et al., 2020). It is a crop of high economic value that is used for food, feed and fuel. This species is the second largest source of seed oil and protein meal, which are major globally traded agricultural commodities (Friedt et al., 2018). Due to the high responsiveness of some genotypes to microspore embryogenesis, *B. napus* is also highly valuable in basic research as a model organism for studying various biochemical and physiological processes occurring during plant embryogenesis and development (Malik et al., 2007). Moreover, the reprogramming of rapeseed microspores can be done solely by heat stress without the addition of plant hormones or other stimulants, which enables direct regeneration without an intervening callus phase. By modulating the duration of heat stress, it is even possible to stimulate the formation of different embryogenic structures, such as compact embryos lacking a suspensor, embryos with suspensors or embryogenic callus-like structures (Joosen et al., 2007; Li et al., 2014; Corral-Martínez et al., 2020). The suspensor-bearing microspore embryogenesis culture system is highly similar to the zygotic embryogenesis pathway since it follows the same developmental pattern. It begins with the formation of a short suspensor, which is followed by the establishment of the embryo-proper cell and later by the formation of a haploid embryo through the same ordered pattern of cell division as in zygotic embryos (Joosen et al., 2007; Supena et al., 2008; Prem et al., 2012).

Phytosulfokine-α (PSK-α) is a small sulfonated pentapeptide synthesized from 80–120 amino acid precursor proteins encoded by small *PSK* gene families (Lorbiecke and Sauter, 2002; Sauter, 2015). It was first identified in conditioned media of *Asparagus officinalis* suspension cells as a stimulant of proliferation at a low cell density that normally would not divide (Matsubayashi and Sakagami, 1996). Later, its *in vivo* activity as an intercellular signal peptide and autocrine growth factor was demonstrated in various plant species and biological processes (Yang et al., 1999; Yang et al., 2001; Lorbiecke and Sauter, 2002; Sauter, 2015). It is ubiquitously present in higher plants and is involved in several developmental processes *in vivo*, such as the response to abiotic and biotic stress, tissue differentiation, and sexual reproduction. Gametophytic and early sporophytic development in maize has been shown to be dependent on PSK availability, which is tightly controlled through differential expression of *PSK* genes in a cell type-specific manner (Lorbiecke et al., 2005). *PSK* genes in maize are expressed in both female and male gametophytes. All four of them (*ZmPSK* 1–4) are expressed in the female gametophyte cells and developing kernels, while *ZmPSK1* and *ZmPSK3* were the only *PSK* paralogues expressed in the male gametophyte and the expression patterns differed between the two genes. Transcripts of *ZmPSK1* and *ZmPSK3* were also found at high levels in secretory tapetal cells of anthers, which supports developing microspores. The predicted functions of these two paralogues were therefore to ensure coordinated mitotic divisions among all microspores contained in one loculus, coordinated pollen wall synthesis or both. PSK also promoted pollen tube growth along the transmitting tract and guided the pollen tube from the transmitting tract to the embryo sac (Lorbiecke et al., 2005). The stimulatory effect of PSK on pollen tube growth was later confirmed in experiments on the *in vitro* pollen tube growth of *Arabidopsis thaliana* and pear (Stührwohldt et al., 2015; Kou et al., 2020). The role of PSK-α in *in vitro* cultures has been studied in the past mostly on protoplast cultures and somatic embryogenesis, as reviewed by Dubas et al (Dubas et al., 2021). There is only one report on the use of exogenous PSK-α in wheat and triticale androgenesis, demonstrating its positive effect on the number of regenerated embryos and green plant production (Asif et al., 2014). However, to the best of our knowledge, there are no reports on gene expression analysis of the effect of exogenously added PSK in *in vitro* plant cultures.

RNA sequencing (RNA-Seq) is a widely used technique in plant research to analyze the expression of either protein coding or non-coding genes or small RNAs. It allows researchers to identify and quantify the expression of genes in plants under different conditions, such as during development, in response to environmental stress, or in response to treatments with chemicals or drugs. It provides a detailed and quantitative overview of gene expression, alternative splicing and allele-specific expression. RNA-Seq analysis of isolated microspores has been performed to identify gene expression in wheat (Seifert et al., 2016), barley (Bélanger et al., 2018), soybean (Hale et al., 2020), rapeseed (Corral-Martínez et al., 2020) and cabbage (Kong et al., 2022).

The aim of our study was to evaluate the effect of exogenously added PSK-α on the viability and haploid embryogenesis of rapeseed microspores and to analyze its influence on their transcriptomes. Transcriptome profiling was performed with next-generation RNA-Seq of 15 RNA libraries obtained from microspore cultures of *Brassica napus* ‘Topaz’ DH4079 cultured in standard NLN-13 medium with or without the addition of 0.1 µM PSK-α (D2+PSK and D2-PSK, respectively) at three time points. The RNA-Seq results were analyzed for differential expression of genes during microspore embryogenesis caused by PSK-α, for the expression of genes involved in the synthesis of PSK and PSK receptors, and for the expression of genes involved in microspore embryogenesis.

## 2 Materials and methods

### 2.1 Microspore culture

Microspore isolation was performed according to the protocol described by Custers (Custers, 2003), with minor modifications, as follows. Briefly, plants of summer rapeseed *Brassica napus* ‘Topaz’ doubled haploid line DH4079 were grown at a constant temperature of 18°C in a controlled environment room with a 16 h photoperiod and a relative humidity of 55–60%. After the first few flowers opened, the inflorescences were collected, and the flower buds were sorted based on their lengths using a stereo microscope. Flower buds between 2.1 and 2.4 mm in length were used for the isolation of microspores since they contained mostly microspores at the late uninucleate stage. The developmental stage of microspores was verified with DAPI staining according to Custers (Custers, 2003).

The isolation of microspores was performed with pre-cooled tools in NLN-13 medium (Lichter, 1982) (Duchefa Biochemie B.V., The Netherlands) and centrifugation steps at 4°C. The isolated microspores were resuspended in pre-cooled NLN-13 medium at density of 2, 4 or 8 × 10^4^ microspores per milliliter. The different culture densities were prepared from the initial suspension of isolated microspores which had its density determined using a counting chamber. The heat shock treatment of microspores was done at 32°C for 48 hours in the dark, which was followed by incubation at 25°C again in the dark. After 7 days of culture (2 days at 32°C followed by 5 days at 25°C), the developing embryos were diluted with fresh NLN-13 medium and after 14 days, cultures in all Petri dishes were diluted 5 times with fresh NLN-13 medium. Four weeks after microspore isolation, the number of embryos was counted per Petri dish. For each treatment, seven petri dishes containing 3 mL of microspore suspensions were prepared, and the experiments were repeated three times.

The viability of the microspores and developing structures was determined with FDA staining (final concentration 1 µg/mL) on the day of microspore isolation and after one week of culture.

### 2.2 Phytosulfokine-α

Phytosulfokine-α (PSK-α) was purchased from PeptaNova GmbH (Germany) as a lyophilized powder (0.1 mg), which was resolved with distilled water to a concentration of 0.1 mM. The stock solution was diluted in sterile NLN-13 culture medium to final concentrations of 0.01, 0.1 and 1 µM. Microspores and developing embryos were cultured in these media from the initiation of microspore culture (day 0) until the end of the experiments, four weeks after microspore isolation.

### 2.3 Transcriptome analysis of cultured microspores

Five different cell type samples were collected for gene expression analysis in three biological replicates, as follows: D0: freshly isolated microspores; D2-PSK: microspores after 48 hours of incubation at 32°C in NLN-13 medium; D2+PSK: microspores after 48 hours of incubation at 32°C in NLN-13 medium supplemented with 0.1 µM PSK; D4-PSK: microspores after 48 hours of incubation at 32°C followed by 48 hours of culture at 25°C in NLN-13 medium; D4+PSK: microspores after 48 hours of incubation at 32°C followed by 48 hours of culture at 25°C in NLN-13 medium supplemented with 0.1 µM PSK.

The microspores were collected by centrifugation of 3 ml suspension cultures with microspore density of 4 × 10^4^ microspores per milliliter at 100 ×g for 3 minutes. Then, the supernatant was discarded, and the pellet was frozen in liquid nitrogen and stored at −80°C until RNA isolation. The total RNA was isolated from approximately 120,000 microspores using a Monarch Total RNA Miniprep Kit (NEB). It was quantified using Nano Drop (Thermo Scientific™), and its integrity was analyzed with an Agilent 2100 Bioanalyzer Eukaryote Total RNA Pico (Agilent Technologies, Inc.). Sequencing of RNA was performed at Novogene (UK) using the mRNA sequencing service and the 150 bp paired-end module. The data were delivered through a cloud service in FASTQ format. RNA-Seq analysis was performed with CLC Genomic Workbench and Server (version 24) (Qiagen) which is implementing the DESeq2 algorithm. Sequencing data were quality checked by QC for Read Mapping and then mapped to *Brassica napus* reference genome version 4.1 (http://brassicadb.cn) and *Brassica napus* reference genome Da-Ae (Ref Seq: GCF_020379485.1, NCBI) using the CLC RNA-Seq Analysis pipeline tool. The DEGs were assessed by filtering mapped genes for a ‘Fold change’ absolute value above 2 and an ‘FDR p-value’ lower or equal to 0.05. Expression analysis of selected genes (*PSK*, *PSKR*, *PSI*, embryogenesis related) was performed by identifying their corresponding *B. napus* genes with BLAST and evaluating their expression values normalized to transcripts per million (TPM). Gene expression analysis was done in CLC, and data visualization was performed with freely available web servers Heatmapper (Babicki et al., 2016) and Venny (Oliveros, 2023). Gene ontology (GO) analysis was performed on GO terms using the R package topGO (version 2.40.0) (Alexa and Rahnenfuhrer, 2023) to show which GO terms were significantly enriched. Coding sequences (CDS) of proteins from the genome Da-Ae (Ref Seq: GCF_020379485.1, NCBI) were annotated using EggNOG v5.0 (Huerta-Cepas et al., 2019). Thus, we obtained 52618 CDS annotated with GO terms, and the list of those genes were used as a background list against which a list of upregulated and downregulated genes were tested in GO enrichment analysis with topGO. TopGO algorithm “weight01” and fisher statistical test were used to obtain enriched GO terms of biological processes, molecular functions and cellular components for up- and downregulated genes, respectively. For significantly enriched GO terms, association with each other and summarization by removing redundant GO terms were performed using the Revigo tool (Supek et al., 2011), and the results of both analyses were visualized using Cytoscape (Shannon et al., 1971). Afterwards, enriched terms and their corresponding enrichment p-values were used in REVIGO online tool (Supek et al., 2011) to remove redundant terms (remove obsolete GO terms: Yes, size of resulting list: 0.7, semantic similarity measure: SimRel), summarize, and visualize obtained enriched terms based on entre UniProt database.

### 2.4 Statistical analysis

Data on the viability of microspores and the number of regenerated embryos were analyzed with Microsoft Excel, and ANOVA and Duncan’s multiple range test were performed using the statistical software program R version 4.2.2 (R Core Team, 2022).

## 3 Results and discussion

### 3.1 PSK-α enhances microspore viability in culture

**Figure 1:**
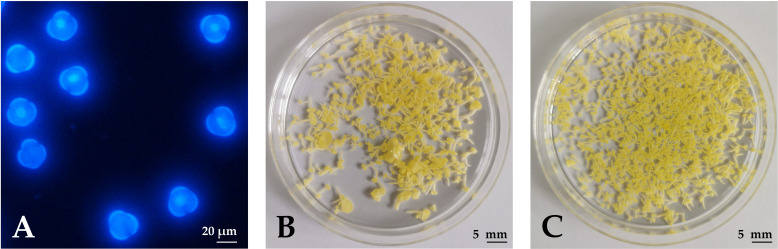
Haploid embryogenesis of rapeseed microspores. **(A)** Microspores under UV epifluorescence (DAPI staining) on the day of isolation when cultures contained uninucleate microspores. **(B)** Developing embryos after 4 weeks of culture in NLN-13. **(C)** Developing embryos after 4 weeks of culture in NLN-13 supplemented with 0.1 µM PSK.

Microspores were isolated after the first 1–3 flowers of the inflorescences opened. Whole inflorescences were sampled and flower buds of appropriate length were used for microspore isolation at the mid- to late-uninucleate stage (**Figure 1A**), which has been demonstrated as the optimal stage for the induction of haploid embryogenesis from micropores (Custers, 2003). During the first days of culture, some of the microspores were enlarged and changed morphology, indicating a switch from the gametophytic to the sporophytic developmental pathway. The viability of the microspores and the developing multicellular structures was evaluated with fluorescein diacetate (FDA) staining and counting green-fluorescing units under an epifluorescent microscope. After one week of culture (2 days at 32°C, followed by 5 days at 25°C), half of the structures, on average, were viable, showing bright green fluorescence.

**Figure 2:**
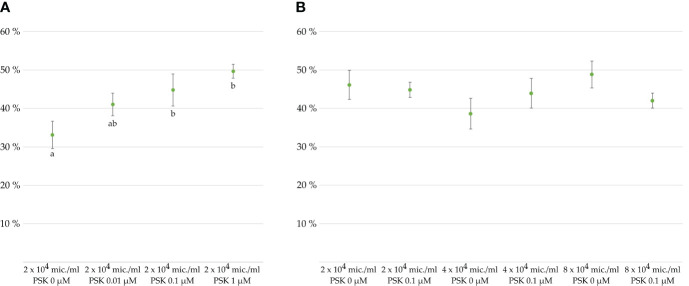
Viability of microspores and developing multicellular structures in percentages after 7 days of culture, as determined with FDA staining. Cultures of microspores **(A)** at a density of 2 × 10^4^ microspores/mL of NLN-13 medium supplemented with various PSK concentrations or **(B)** at different microspore densities in NLN-13 medium supplemented or not with 0.1 µM PSK.

To verify the effect of PSK-α (further referred to simply as PSK) on the viability of microspores and developing embryos, we cultured microspores in different culture media supplemented or not with PSK. The control medium was composed of NLN-13 alone, while PSK was added to the three other tested media in concentrations of 0.01, 0.1 or 1 µM PSK. The differences between microspore viability after 1 week of culture in different media were marginally statistically significant (p = 0.037), with the lowest viability recorded for microspores cultured in NLN-13 alone (33.1 ± 3.6%). The highest viability was recorded in NLN-13 supplemented with 1 µM PSK (49.7 ± 1.8%), but it was not significantly different from the viability of microspores in NLN-13 supplemented with 0.1 µM PSK (44.8 ± 4.2%) (**Figure 2A**). Therefore, we decided to continue our experiments by culturing microspores in NLN-13 supplemented with 0.1 µM PSK, which was also demonstrated to be the optimal concentration of PSK for stimulating the division of *Brassica* protoplasts (Kiełkowska and Adamus, 2017, 2019), also in our recent study (Vogrinčič et al., 2024).

In the following experiments, we tested the effect of 0.1 µM PSK on the viability of microspores cultured at different culture densities: optimal density of 4 × 10^4^ microspores/mL (Custers, 2003), lower than optimal density of 2 × 10^4^ microspores/mL, and higher than optimal density of 8 × 10^4^ microspores/mL. A multifactor analysis of variance (ANOVA) of three replicates per treatment revealed no statistically significant differences between the viability of microspores cultured in NLN-13 alone or in NLN-13 supplemented with 0.1 µM PSK (p = 0.733) or at different densities of microspores (p = 0.375). The interaction between the variables was also not significantly different (p = 0.226) (**Figure 2B**).

### 3.2 PSK stimulates microspore embryogenesis

**Figure 3:**
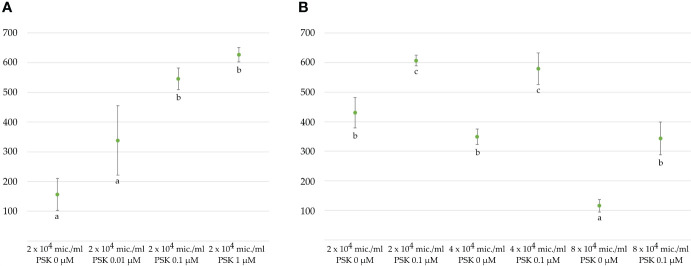
Average number of regenerated embryos per 3 mL microspore culture after 4 weeks **(A)** in NLN-13 medium supplemented with various concentrations of PSK or **(B)** at different initial microspore densities in NLN-13 medium supplemented or not with 0.1 µM PSK.

Microspores and multicellular structures were cultured further at 25°C and embryos developed by the fourth week of culture when regenerated embryos were counted in three replicates of 3 mL cultures (**Figures 1B, C**). The ANOVA showed a statistically significant (p < 0.001) effect of PSK on the number of regenerated embryos, with the highest number of regenerated embryos (626.8 ± 24.5) in the NLN-13 medium supplemented with 1 µM PSK and the lowest number in the NLN-13 medium without PSK (156.2 ± 54.4; **Figure 3A**). In addition, the following experiment demonstrated that supplementing NLN-13 with 0.1 µM PSK had a significant effect at all three tested culture densities of 2, 4 and 8 × 10^4^ microspores per milliliter (**Figure 3B**). In none of the experiments were morphological differences observed between embryos developing in control medium or in medium supplemented with PSK. Multifactor ANOVA showed that both the density of microspores in culture and the addition of PSK had a statistically significant effect, with p values below 0.001 for both variables (**Figure 3B**).

Our results demonstrated a positive effect of PSK on the viability of microspores in culture, on their responsiveness to deviate from the gametophytic pathway and to enter the sporophytic pathway, resulting in haploid and doubled haploid embryo regeneration. Based on our experimental data, we cannot conclude whether it is due to prolonged viability of isolated microspores or to stimulation of progression of the cell division cycle, as was observed in non-embryogenic cell cultures of carrot (Eun et al., 2003). Several reports have already been published on the stimulatory effect of PSK in *in vitro* cultures but mostly for protoplast cultures (Grzebelus et al., 2012; Kiełkowska and Adamus, 2019; Vogrinčič et al., 2024) and somatic embryogenesis (Hanai et al., 2000; Igasaki et al., 2003; Umehara et al., 2005; Wu et al., 2019; Yang et al., 2020). However, to the best of our knowledge, only one scientific article has described the use of PSK for microspore embryogenesis (Asif et al., 2014). In wheat and triticale, concentrations from 0.005 to 0.1 µM were tested along with the control, and the highest concentration of 0.1 µM the most efficient, stimulating the formation of a large number of embryos and green plant regeneration. Our results in *B. napus* confirmed those from wheat and triticale and additionally demonstrated that even higher concentrations of PSK can be stimulatory. The highest concentration tested in our experiments was 10 times higher than in Asif et al (Asif et al., 2014), and this concentration of PSK (1 µM) stimulated the production of the highest number of embryos. This result opens new possibilities for the optimization of haploid induction since even higher concentrations could be tested to exploit the maximal potential of exogenously added PSK in culture media to induce haploid embryogenesis.

### 3.3 Transcriptome profiling of microspores cultured with or without the addition of PSK

Since PSK showed a positive effect on microspore viability and responsiveness to haploid embryogenesis, we sequenced the transcriptomes of microspores in the early stages of culture. Total RNA was isolated from microspores on the day of isolation (D0), after a 48-hour heat-shock induction treatment (D2) and after a further 2 days of culture at 25°C (D4). At days two and four of microspore culture, RNA was isolated from 3 mL cultures of microspores cultured in NLN-13 medium without PSK (D2-PSK, D4-PSK) or with the addition of 0.1 µM PSK (D2+PSK, D4+PSK). RNA was isolated and sequenced from three biological replicates for each treatment.

The obtained sequences were mapped to *Brassica napus* genome version 4.1 (http://brassicadb.cn) and the chromosome-level assembled and annotated *Brassica napus* reference genome Da-Ae (Ref Seq: GCF_020379485.1, NCBI). A comparison of mapping results revealed that a higher number (percentage) of reads mapped to the Da-Ae genome (**Supplementary Table S1**) with more than 95% of reads successfully mapped to 123,215 genes. This clearly confirmed its better completeness, so we decided to use the Da-Ae genome in further analyses, although 4.1 was used in previous studies on rapeseed transcriptome analyses (Yang et al., 2018; Corral-Martínez et al., 2020; Yu et al., 2022).

**Figure 4:**
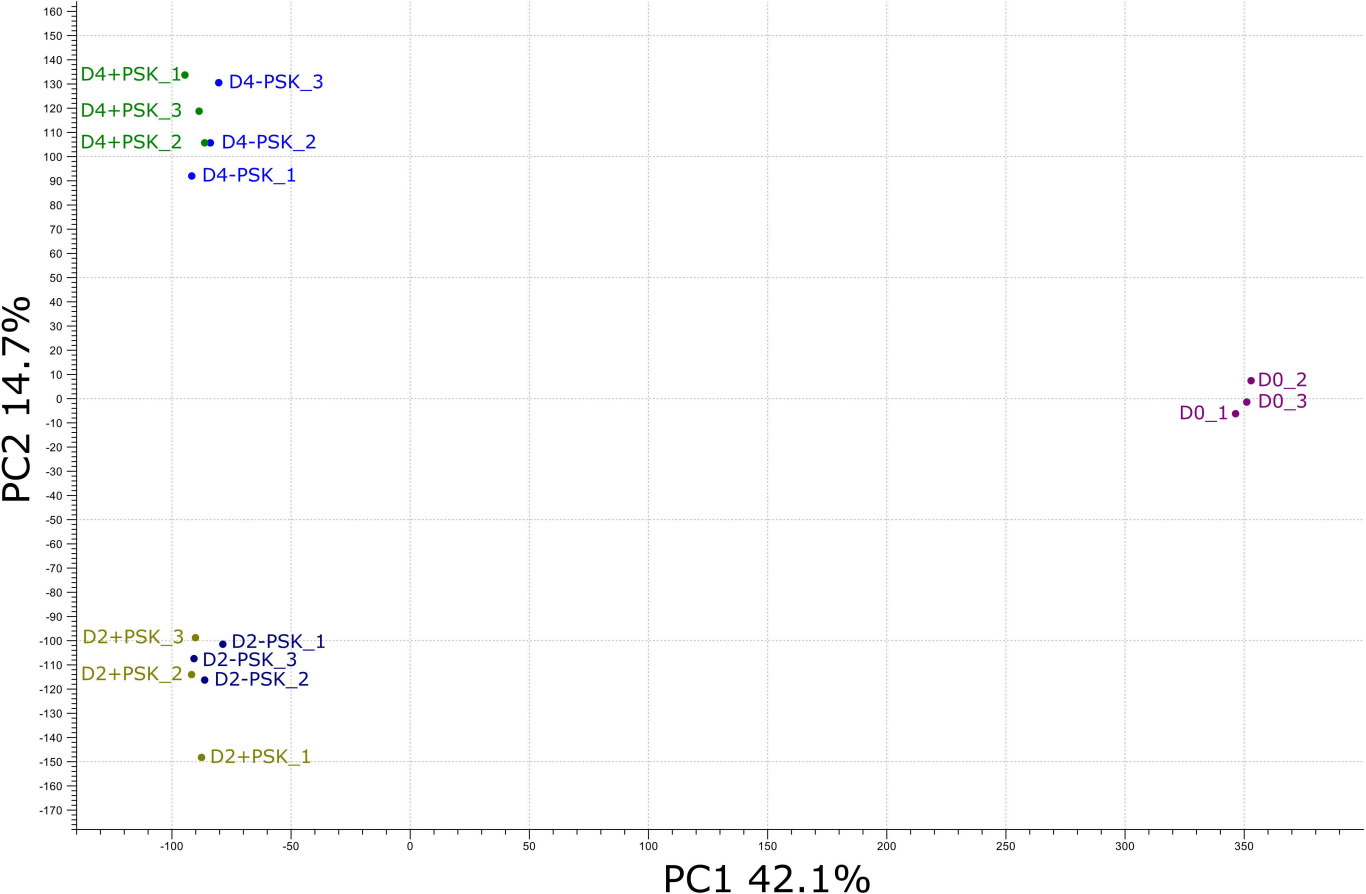
PCA of the RNA-Seq expression data from different cultures on the day of microspore isolation (D0) and after two (D2) and four (D4) days of culture. Transcriptome sequencing at days two and four was performed for microspores cultured in NLN-13 medium with (D2+PSK, D4+PSK) or without (D2-PSK, D4-PSK) the addition of 0.1 µM PSK.

After mapping, a principal component analysis (PCA) of the RNA-Seq expression data was performed. The analysis grouped the transcriptome data into three groups. The first group was composed of sequences from three biological replicates of freshly isolated microspores (D0_1, D0_2 and D0_3, **Figure 4**). In the second group were transcripts from two-day-old microspore cultures and in the third group from 4-day-old cultures, again three replicates per time point and treatment. PCA analysis showed that samples from the same time point had similar transcriptome profiles, which were distinct from the transcriptome profiles at the other two time points. The first two principal components explained 56.8% of the observed variability in gene expression between different samples and time points, with PC1 explaining 42.1% and PC2 explaining 14.7% of the variability. PC1 discriminated the transcriptomes of microspores grown *in vivo* (D0) from those in culture (D2 and D4), while PC2 discriminated microspores at different time points of culture (D2 or D4) when, in addition to microspores, multicellular structures at different developmental stages were present. Although there were some differences among the transcriptomes of microspore cultures with or without the addition of PSK in the PCA plot, these differences were comparable to the differences observed between the biological replicates of the same treatment.

Filtering mapped sequences for expression values above 0 (TPM) in all three replicates enabled us to compare the expressed sequences between our treatments. The total number of expressed genes per treatment was: 52,197 in D0; 45,602 in D2-PSK; 44,436 in D2+PSK; 47,435 in D4-PSK; and 47,161 in D4+PSK. The proportions of uniquely expressed genes varied between 1.2 and 16.1%, depending on the compared treatments. The results of all comparisons are shown in **Supplementary Figure 1**.

### 3.4 Differential expression profiling of microspores during the early stages of culture in PSK-supplemented medium

The RNA-Seq results were further analyzed for differentially expressed genes in microspore samples cultured with or without PSK in the culture medium. Significant DEGs were considered genes with FDR p-values lower or equal to 0.05 and an absolute fold change above 2.

On the second day of culture at 32°C in PSK-supplemented medium, there were 45 DEGs, 15 of which were upregulated and 30 of which were downregulated compared to the transcriptomes of microspores in NLN-13 alone (**Table 1**). The highest differences in expression were recorded for genes *rac-like GTP-binding protein RHO1* (LOC125596755, +237.99), *cathepsin B-like protease 2* (LOC125596607, +133.61) and *GDSL esterase/lipase At3g09930-like* (LOC125609099, +102.37). After a further 2 days of culture at 25°C, the number of DEGs increased to 304 and again a higher number (274) of genes showed downregulation (**Table 1**). The highest differences were recorded for *heptahelical transmembrane protein 4* (LOC125603703, +1359.28), and *choline-phosphate cytidylyltransferase 2-like* (LOC125575084, −108.03). However, some of the genes showed no detectable expression in microspores cultured in the standard NLN-13 medium, resulting in an expression value of 0 for control samples. Fold change and Log_2_ fold change values, calculated to assess such differential expression, were excluded from the lists of DEG in **Table 1**.

**Table 1:** Number of differentially expressed genes (DEGs) in microspores at three time points of culture in various culture media.

Timepoint	PSK (µM)	No of DEGs	No of upregulated genes	No of downregulated genes	Fold change
Day 2	0.1 vs. 0	45	15	30	From −37.32 to +237.99
Day 4	0.1 vs. 0	304	30	274	From −108.03 to +1,359.28
Day 2 vs. Day 0	0	28,345	11,393	16,952	From −1,720.96 to +49,883.10
Day 2 vs. Day 0	0.1	27,866	11,279	16,587	From −1,633.36 to +52,101.21
Day 4 vs. Day 0	0	28,731	11,746	16,985	From −3,195.41 to +75,996.53
Day 4 vs. Day 0	0.1	29,245	11,907	17,338	From −2,311.51 to +72,435.57

RNA was isolated from microspores on the day of microspore isolation, after 2 and 4 days of microspore culture in NLN-13 medium supplemented or not with 0.1 µM PSK.

The table presents the counts of DEG and their respective fold changes, excluding genes with a zero-expression value in the control samples.

The list and corresponding statistics of all significant DEGs in microspore samples cultured with or without the addition of 0.1 µM PSK are presented in **Supplementary Tables S2**, **S3**.

By comparing the DEGs in both media at days 2 and 4, we identified 20 DEGs at both time points when PSK was added to NLN-13. The vast majority (18) showed downregulation at both time points, while two showed upregulation at one time point and downregulation at the other. The gene *40S ribosomal protein S3-2-like* (LOC106453640) was downregulated on day 2 (−5.45) and upregulated on day 4 (+3.76), while the gene *U-box domain-containing protein 35-like* (LOC125608832) was upregulated on day 2 (+38.76) and downregulated on day 4 (−67.94). The list of genes with corresponding statistics is presented in **Table 2**.

**Table 2:** List of differentially expressed genes (DEGs) on the second and the fourth day of microspore culture in medium with or without 0.1 µM PSK.

Gene symbol	Fold change on day 2	Fold change on day 4	NCBI description for *Brassica napus*
LOC106453640	−5.45	3.76	40S ribosomal protein S3-2-like
LOC106389644	−2.41	−2.02	non-specific lipid transfer protein GPI-anchored 1
LOC106425829	−2.75	−2.10	leucine-rich repeat protein 1
LOC106347881	−2.39	−2.20	peroxidase 42
LOC111197712	−3.46	−2.48	glutamine synthetase cytosolic isozyme 1-2
LOC106386441	−2.30	−2.58	GDSL esterase/lipase At5g45670
LOC106370389	−5.48	−2.97	uncharacterized
LOC106376949	−2.81	−3.20	ATP-dependent RNA helicase A
LOC106375370	−4.72	−3.36	GDSL esterase/lipase At5g45670
LOC125589932	−9.51	−3.50	non-specific lipid-transfer protein 5-like
LOC106442906	−3.64	−3.66	follicular dendritic cell secreted peptide
LOC106439790	−5.51	−3.74	glycine-rich protein 3 short isoform
LOC106357039	−8.73	−4.64	GDSL esterase/lipase At1g71691
LOC125585666	−4.98	−4.78	non-specific lipid-transfer protein B
LOC106361471	−11.85	−5.15	proline-rich protein 4
LOC106376871	−4.07	−5.51	non-specific lipid-transfer protein 5-like
LOC106426754	−7.21	−9.58	glycine-rich protein 3 short isoform
LOC125588812	−8.13	−12.44	probable LRR receptor-like serine/threonine-protein kinase At1g51820
LOC125581325	−8.26	−17.87	squalene epoxidase 5-like
LOC125608832	38.76	−67.94	U-box domain-containing protein 35-like

Fold changes preceded by + denotes upregulation and those preceded by – downregulation of genes when comparing transcriptomes in PSK supplemented medium with transcriptomes in regular NLN-13 culture medium.

We further compared the expression of genes on days two and four to the expression of genes on the day of microspore isolation (**Table 1**). Microspores at day 2 altered the expression of 28,345 genes in NLN-13 and 27,866 genes in NLN-13 supplemented with PSK. Among the DEG, 25,597 were differentially expressed under both culture conditions, 2,748 were differentially expressed only when comparing D2-PSK to D0 and 2,269 were differentially expressed only when comparing D2+PSK with D0.

On day four, 29,245 genes were differentially expressed in the cultures supplemented with PSK compared to D0, and 28,731 genes were differentially expressed in the cultures without the addition of PSK. Among the DEG, 26,282 were differentially expressed under both culture conditions; 2449 were differentially expressed only when comparing D4-PSK to D0 and 2963 were differentially expressed only when comparing D4+PSK to D0. With the duration of microspore culture, the number of differentially expressed genes and the range of differential expression increased (**Table 1**).

We could not compare our differential profiling with other reports since the only published RNA-Seq analysis of *B. napus* microspore cultures was aimed at elucidating the cell fate identity of embryogenic callus (Corral-Martínez et al., 2020) and not on the analysis of DEGs.

### 3.5 Effect of PSK on metabolic pathways in microspore embryogenesis

**Figure 5:**
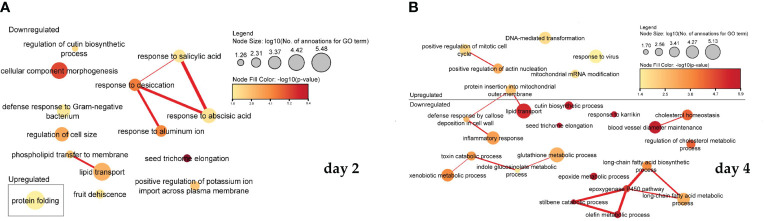
Association of significantly enriched gene ontology (GO) terms containing differentially expressed genes involved in biological processes on the second **(A)** and fourth **(B)** day of microspore culture. Node sizes represent the logarithm of the number of genes belonging to the GO terms, and the color scale represents the negative logarithm of the *p*-value of the enriched GO terms. The width of the lines denotes the degree of semantic similarity between enriched GO terms (information content, e.g., the frequencies of two GO terms and their closest common ancestor term). The similarity of the processes and the association between them in the expression of the genes belonging to the term are encoded by the thickness of the connecting lines.

In the process of ME, the addition of PSK activated some and inhibited other biological signalling pathways involved in cell regeneration, microspore formation and development into mature embryos, and further plant regeneration. Interestingly, the key processes differed depending on whether the RNA was sampled after two or four days. After two days, most processes were downregulated, and after four days of treatment with PSK, the processes involved in cell formation, cell division, growth and regeneration began to be actively expressed. Statistically significant results (weight algorithm and Fisher statistical test; *p*-value < 0.05) showed that they were involved in various biological processes, mostly as a result of reduced gene activity. The resulting biological processes are shown in **Figure 5A** for day 2 and in **Figure 5B** for day 4.

**Figure 6:**
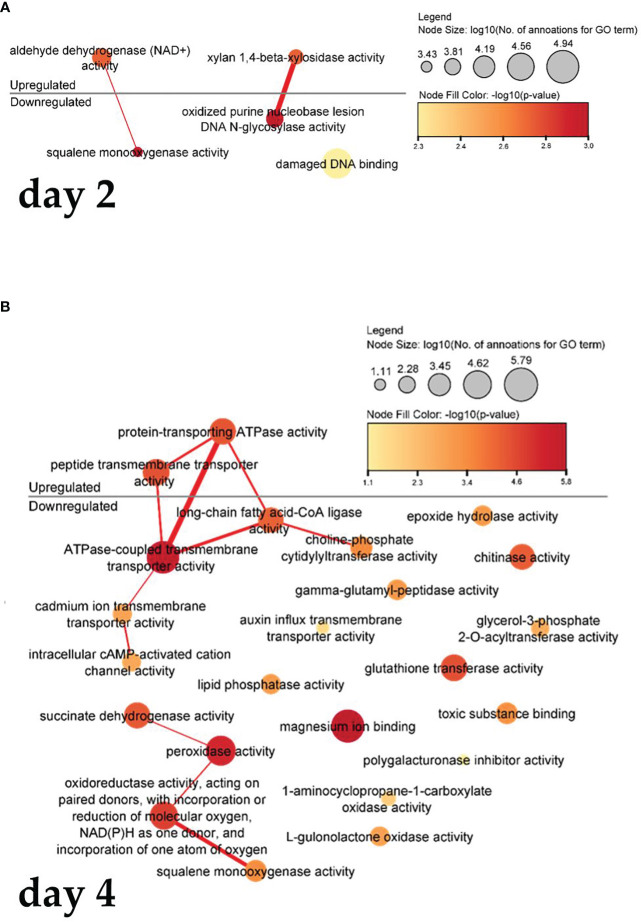
Enriched molecular functions in cells on the second **(A)** and fourth **(B)** day after treatment. Node sizes represent the logarithm of the number of genes belonging to the GO terms, and the color scale represents the negative logarithm of the *p*-value of the enriched GO terms. The width of the lines denotes the degree of semantic similarity between enriched GO terms (information content, e.g., the frequencies of two GO terms and their closest common ancestor term).

**Figure 6** shows a visualization of the interrelated function of molecules. In cells treated with PSK, after two days (**Figure 6A**), there is increased gene expression of the activity of adenine dehydrogenase (NAD+), which is used in the electron transport chain to generate ATP, resulting in decreased activity of squalene monooxygenase activity, as the latter activity is related to the binding and amount of NADPH in the cells. Xylan-1,4-beta-xylosidase activity and oxidized purine nucleobase lesion activity of DNA N-glycosylates also act synergistically.

Four days after treatment with PSK (**Figure 6B**), protein-transporting ATPase activity and transmembrane peptide transport activity increased, leading to a decrease in ATPase-coupled, cadmium ion, intracellular cAMP-activated cation channel activity and a reduction in the activity of long-chain fatty acid CoA ligase, which catalyzes the precursor reaction for β-oxidation of fatty acids and can be incorporated into phospholipids. After four days in culture, there was decreased expression of succinate dehydrogenase activity and peroxidase activity, whose function is to degrade hydrogen peroxide, one of the toxins produced as a byproduct of using oxygen for respiration. The following were also downregulated: oxidoreductase activity, which acts on paired donors, incorporation or reduction of molecular oxygen, NAD(P)H as a donor, incorporation of an oxygen atom, and squalene monooxygenase activity (also downregulated two days after treatment).

### 3.6 Expression of *PSK*, *PSKR* and *PSI* genes

To verify the expression of genes responsible for the synthesis of PSK, PSK receptors and PSK simulators, we performed BLAST analysis of protein sequences from *Arabidopsis thaliana*: six PSK protein sequences (PSK 1, 2, 3, 4, 5, 6), two PSK receptor protein sequences (PSKR 1, 2) and three PSK simulator protein sequences (PSI 1, 2, 3). For each of these sequences, more than one and up to four high-scoring segment pairs (HSP) with similarities above 70% were obtained and included in the analysis (**Supplementary Table S4**). Most of the 35 genes selected based on identity scores were identified as *B. napus PSK*, *PSKR* and *PSI* genes in NCBI. However, some of them were annotated differently in *B. napu*s compared to *A. thaliana* annotations. For example, the ortholog of the *A. thaliana AtPSK3* gene was annotated as putative phytosulfokines 6 in *B. napus*. The ortholog of the *AtPSK4* gene was annotated as phytosulfokines 3, and the ortholog of the *AtPSK6* gene was annotated as putative phytosulfokines 4 and VQ motif-containing protein 29-like (**Supplementary Table S4**). We used *A. thaliana* annotations in further interpretation of the results.

**Figure 7:**
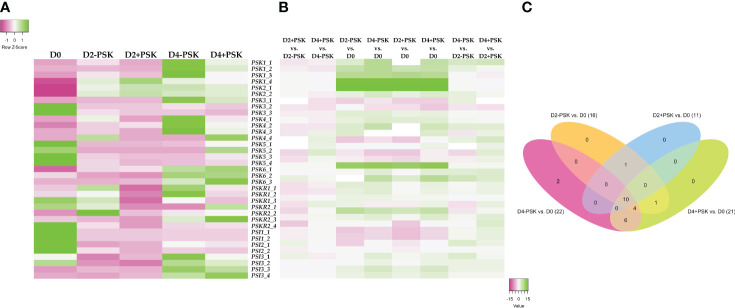
Expression analysis of genes coding for PSK, PSK receptors and PSK simulators. RNA was isolated and sequenced from microspores on the day of isolation (D0), after two days of culture (D2-PSK, D2+PSK) and after four days of culture (D4-PSK, D4+PSK). **(A)** Heatmap of average expression data in TPMs scaled in rows. Expression levels lower than average and higher than average for each gene are marked in pink and green, respectively. **(B)** Heatmap of log fold change. **(C)** Venn diagram of differentially expressed genes in various comparisons.

Comparison of expressed genes and identification of unique ones are presented in **Supplementary Figure 2**. The average expression values of the selected 35 genes from three biological replicates of D0, D2-PSK, D2+PSK, D4-PSK and D4+PSK are presented in **Figure 7**. As shown in the heatmap (**Figure 7A**) and expression results in **Supplementary Table S4**, genes for PSK1 and PSK2 were not expressed in uninucleate microspores on the day of their isolation. This is in contrast to reports in maize, in which high mRNA levels of *ZmPSK1* and *ZmPSK3* were found in uninucleate microspores (Lorbiecke et al., 2005). In our data, higher expression was recorded for *PSK3*, *PSK6* (gene LOC111199764), and proteins PSK SIMULATOR 1 and 2-like. During microspore culture at days 2 and 4, the *PSK2* genes showed the highest increase of expression and the highest overall expression among the *PSK*, *PSKR* and *PSI* genes with TPM values from 76.565 to 198.829. This could indicate the progression of a large part of cultured microspores in their gametophytic development toward pollen maturity since *AtPSK2* showed a strong expression in *A. thaliana* pollen cells (Stührwohldt et al., 2015). It is known from the literature that during microspore culture, most cultured cells either stop dividing or continue their gametophytic development, and only a small proportion of the initial population is successfully reprogrammed toward sporophytic development (Corral-Martínez et al., 2020). This was also reflected in our experiments, where an average of 600 embryos developed from 120,000 cultured microspores, indicating that only about 0.5% of microspores were able to develop into embryos. The others died or continued their gametophytic development. In summary, the overall expression of genes for PSK, PSKR and PSI increased during microspore culture, as indicated by an increase of sum TPM values from 27.009 at D0 to 268.450 (D2-PSK), 306.657 (D2+PSK), 331.584 (D4-PSK) and 300.065 (D4+PSK) (**Supplementary Table S4**).

Although there were differences in the expression of these genes between treatments (D0, D2-PSK, D2+PSK, D4-PSK and D4+PSK), only the gene phytosulfokine 1 (LOC106374539) showed significant differential expression when PSK was added to the culture medium. Its TPM value at day 4 in NLN-13 was 8.6, while with the addition of PSK, its expression dropped to 3.6, showing a 2.43-fold downregulation (−1.28 log2 fold change). The other genes did not show significant differential expression (FDR p-values above 0.05) in microspores cultured with or without PSK. We assumed that the lack of significance was due to the relatively low expression levels in our samples. A graphical representation of the log fold change differences in gene expression is shown in **Figure 7B**, and the respective data are shown in **Supplementary Table S4**.

In contrast, several of the selected genes showed significant differential expression when comparing transcriptomes from different time points. Most genes were upregulated when comparing transcriptomes from days 2 and 4 to day 0. As presented in **Figure 7C** and **Supplementary Tables S5**, 10 showed significant differential expression in all comparisons: *PSK1* (LOC106374539, LOC106434782), *PSK2* (LOC106392126, LOC106449807), *PSK3* (LOC106372650), *PSK6* (LOC106409702), *PSI1* (BNAC06G08150D, LOC106367043) and *PSI3* (LOC106380541, LOC125574825). The results indicate that these genes are significantly upregulated when culturing microspores *in vitro* and inducing them towards microspore embryogenesis.

### 3.7 Expression of embryogenesis-related molecular markers and genes

Previous studies on early events in embryogenesis and embryo maturation lead to the development of potential molecular markers (Malik et al., 2007) and the discovery of several embryogenesis related genes (Perry et al., 1996; Lotan et al., 1998; Friml et al., 2003; Haecker et al., 2004; Lukowitz et al., 2004; Stadler et al., 2005; Joosen et al., 2007; Bayer et al., 2009; Cole et al., 2009; Berger et al., 2011; Ueda et al., 2011; Soriano et al., 2013; Li et al., 2014; Slane et al., 2014; Horstman et al., 2015; Seifert et al., 2016; Corral-Martínez et al., 2020), one of which, the *BABY BOOM* gene, was identified from embryogenic microspore cultures of *B. napus* ‘Topas’ DH 4079 (Boutilier, 2002). In our study, homologous genes from the reference genome Da-Ae were chosen for the analysis of their expression within our RNA-Seq data.

In the study of Malik et al (Malik et al., 2007), EST libraries of rapeseed ME cultures were used for the development of a total of 16 molecular markers belonging to three groups: embryo-specific, embryo-expressed and sporophyte-specific molecular markers. Among the 16 molecular markers, 3 and 15 showed statistically significant differential expression in our PSK-supplemented cultures at days 2 and 4, respectively. Fold change differences varied between −7.14 for *cytochrome P450 78A6* (LOC106402213, D4+PSK vs. D4-PSK) and +8.37 for *napin-like* gene (LOC106445672, D2+PSK vs. D2-PSK). Analyzing each gene (with a unique LOC identifier) separately, only *leucine-rich repeat protein 1* (LOC106425829) showed significant differential expression at both analyzed time points. It was downregulated 2.75 fold and 2.1 fold after 2 and 4 days of culture, respectively, if PSK was added to the culture medium. Other genes were also differentially expressed at both time points but with different LOC identifiers. For example, paralogues for the *sporophyte-specific cytochrome P450* were downregulated at days 2 (LOC106412358) and 4 (LOC106402213, LOC106371512, LOC106369101), while embryo-expressed genes for *napins* were upregulated at days 2 (LOC106445672) and 4 (LOC106430395, LOC106433705, LOC106449756). Except for the *napin* genes, which were upregulated in the presence of PSK, all other markers were downregulated when PSK was exogenously added to the culture medium, which was unexpected based on our results of regenerated embryos.

**Supplementary Table S6** presents the list of differentially expressed embryogenesis-related molecular markers in our cultures with their expression values, which are also presented in the heatmaps in **Supplementary Figure 3**. As shown, the embryo-specific molecular markers were predominantly expressed after 4 days of culture and had higher expression in medium without PSK (samples D4-PSK), while on the day of microspore isolation (D0), numerous sporophyte-specific molecular markers were highly expressed.

The analysis was expanded to all relevant genes involved in embryogenesis based on a literature search (Perry et al., 1996; Lotan et al., 1998; Friml et al., 2003; Haecker et al., 2004; Lukowitz et al., 2004; Stadler et al., 2005; Joosen et al., 2007; Bayer et al., 2009; Cole et al., 2009; Berger et al., 2011; Ueda et al., 2011; Soriano et al., 2013; Li et al., 2014; Slane et al., 2014; Horstman et al., 2015; Seifert et al., 2016; Corral-Martínez et al., 2020). A list of DEGs based on different comparisons is presented in **Supplementary Table S7**. Thirty-three of them were analyzed as embryogenesis-related molecular markers, and 6 of them were not differentially expressed in any of the comparisons (LOC106360444, LOC106394811, LOC106407345, LOC106432034, LOC106439563, LOC106400845).

Three of the embryogenesis-related genes were differentially expressed at day 2, all of which were downregulated in PSK-supplemented medium: *cytochrome P450 78A5-like* (LOC106412358), *follicular dendritic cell secreted peptide* (LOC106442906) and *non-specific lipid-transfer protein B* (LOC125585666). On day 4, 9 genes were differentially expressed, all of which were downregulated. The genes *follicular dendritic cell secreted peptide* (LOC106442906) and *non-specific lipid-transfer protein B* (LOC125585666) were significantly downregulated at both time points in media supplemented with PSK. **Supplementary Table S7** shows a list of differentially expressed embryogenesis-related genes in different comparisons. In all of them, 79 genes were differentially expressed.

## 4 Conclusions

This study presents the first comprehensive analysis of gene expression during ME of *Brassica napus*, a model organism for ME studies. This complements earlier studies on gene expression performed on EST sequencing (Malik et al., 2007) and microarray analyses (Maraschin et al., 2006; Joosen et al., 2007; Muñoz-Amatriaín et al., 2009; Li et al., 2014), which are limited by the type of microarray platform used and therefore cannot cover all genes specifically expressed in the reprogramming process of ME. Using RNA-Seq, we identified DEGs during ME and quantified their expression. Moreover, we demonstrated a stimulatory effect of the mitogenic factor PSK-α on the induction of haploid embryogenesis of cultured microspores. Transcriptome analysis revealed genes that were differentially expressed in PSK-supplemented media, most of which showed downregulation when compared to the transcriptomes of microspores cultured in NLN-13 alone.

The reported results enable a deeper understanding of the molecular mechanism underlying the developmental switch of microspores toward the sporophytic route and further enable the development of new early embryogenesis markers in the future.

## Data availability statement

The RNA sequencing data generated and analyzed within this study are available in the NCBI Sequence Read Archive (SRA) repository (https://www.ncbi.nlm.nih.gov/sra/) under the BioProject ID PRJNA972444 (https://www.ncbi.nlm.nih.gov/bioproject/PRJNA972444).

## Author contributions

ŠMM: Conceptualization, Data curation, Formal analysis, Investigation, Methodology, Writing – original draft. UK: Data curation, Formal analysis, Investigation, Methodology, Writing – original draft, Software. VV: Writing – original draft, Conceptualization. JJ: Conceptualization, Data curation, Formal analysis, Funding acquisition, Methodology, Resources, Software, Supervision, Writing – review & editing. JM: Data curation, Formal analysis, Funding acquisition, Methodology, Resources, Software, Supervision, Writing – review & editing, Conceptualization, Investigation, Project administration, Validation, Visualization, Writing – original draft.
