# Supplementary material for: The effect of phytosulfokine alpha on haploid embryogenesis and gene expression of Brassica napus microspore cultures
Source: Front Plant Sci. 2024 Feb 15;15:1336519. doi: 10.3389/fpls.2024.1336519 (PMC10902448; doi:10.3389/fpls.2024.1336519)

Supplementary Material

Supplementary Figure 1: Comparative analysis of gene expression in different treatments. The analysis was done by filtering mapped sequences for expression values above 0 (TPM) in all three biological replicates per treatment. Under each treatment's designation, the total count of expressed genes per treatment is provided. Circular diagrams visually depict the distribution of expressed genes within each treatment category, with numerical annotations inside the circles indicating the number and percentage of genes expressed in the respective treatment. The overlapping portions of the circles highlight the count of genes expressed in two or all three treatments, offering insights into shared gene expression patterns among different experimental conditions.

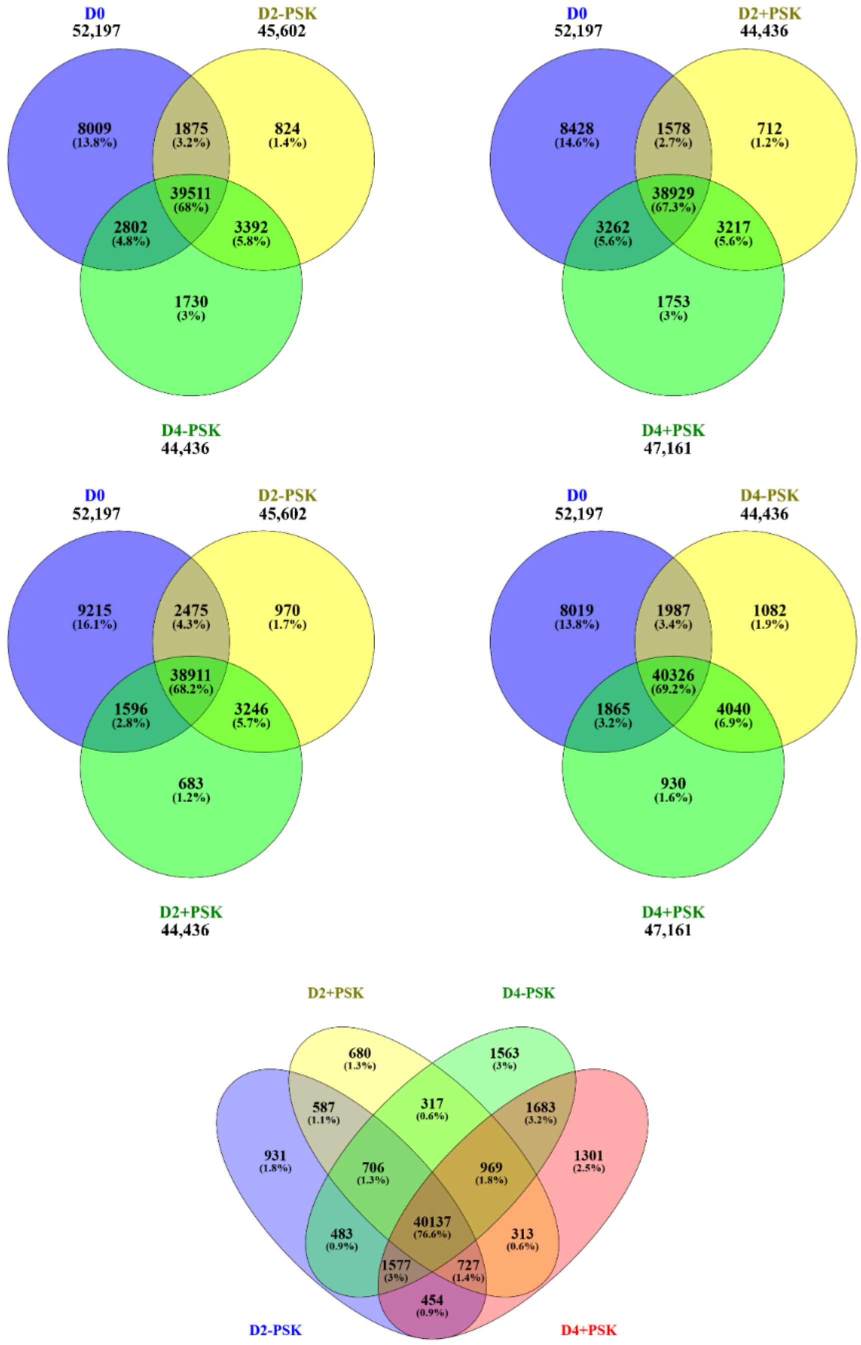

Supplementary Figure 2: Comparative analysis of *PSK*, *PSKR* and *PSI* gene expression in different treatments. The analysis was done by filtering mapped sequences for expression values above 0 (TPM) in all three biological replicates per treatment. Under each treatment's designation, the total count of expressed genes per treatment is provided. Circular diagrams visually depict the distribution of expressed genes within each treatment category, with numerical annotations inside the circles indicating the number and percentage of genes expressed in the respective treatment. The overlapping portions of the circles highlight the count of genes expressed in two or all three treatments, offering insights into shared gene expression patterns among different experimental conditions.

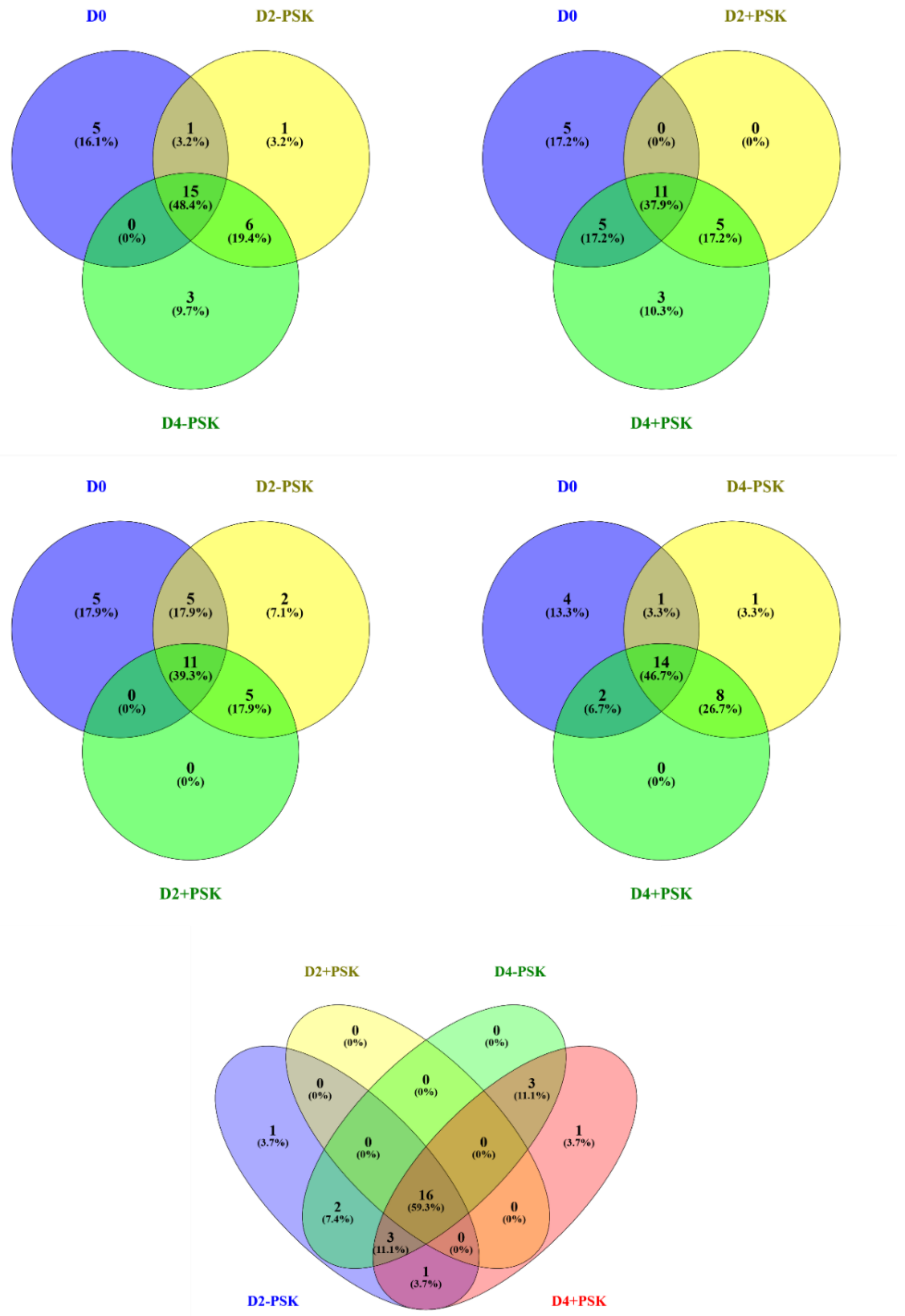

Supplementary Figure 3: Heat maps of average expression data in TPMs of microspore embryogenesis molecular markers developed by Malik et al., 2007 from EST libraries. Markers are grouped based on their expression specificity into three groups: embryo specific, embryo expressed and sporophyte specific molecular markers. Values are scaled in rows.

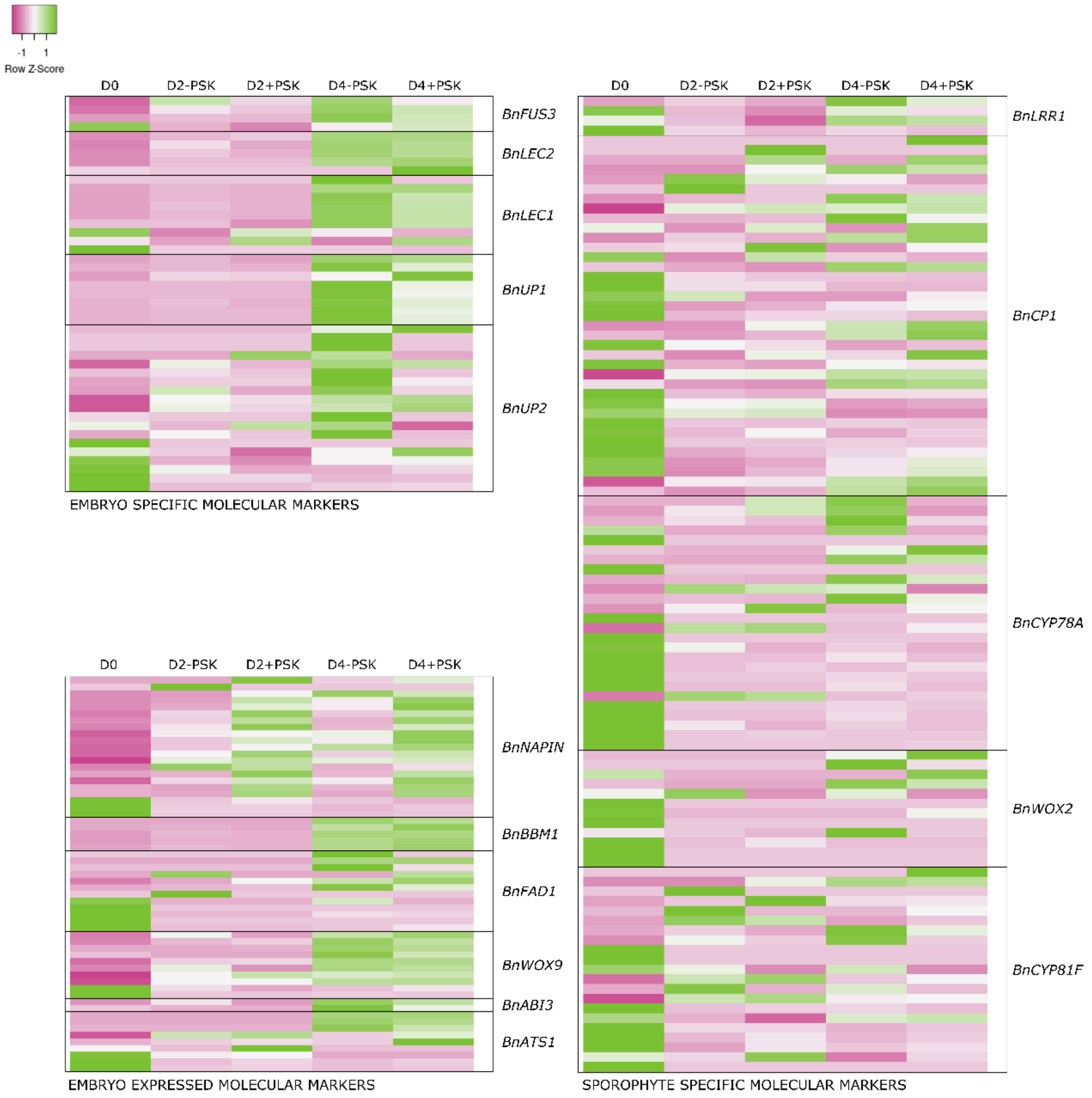

Supplement: Supplementary file 1 [file Image_1.pdf]
